# Importance of Health Aspects in Polish Consumer Choices of Dairy Products

**DOI:** 10.3390/nu10081007

**Published:** 2018-08-02

**Authors:** Marta Sajdakowska, Jerzy Gębski, Krystyna Gutkowska, Sylwia Żakowska-Biemans

**Affiliations:** Department of Organization and Consumption Economics, Faculty of Human Nutrition and Consumer Sciences, Warsaw University of Life Sciences (SGGW-WULS), 159C Nowoursynowska Street, 02-787 Warsaw, Poland; jerzy_gebski@sggw.pl (J.G.); krystyna_gutkowska@sggw.pl (K.G.); sylwia_zakowska_biemans@sggw.pl (S.Ż.-B.)

**Keywords:** consumer, dairy products, health aspects

## Abstract

In general, dairy products are well regarded for their nutritional value. Consumer perception of dairy products is influenced by many interrelated factors but healthiness remains one of the key attributes and values for consumers. Furthermore, contemporary consumers increasingly seek out dairy products with additional health benefits and, therefore, it is essential to explore which attributes are important drivers of food choices and how producers can better respond to shifting consumer values and needs in each dairy product category. Therefore, the aims of the study were: (a) to identify consumer segments based on the importance they attached to selected attributes of dairy products, (b) to explore differences between the identified segments in their perceptions of health-related attributes of dairy products, (c) to determine if health-related aspects influenced consumers decisions to buy high-quality dairy products, and (d) to identify if consumers were open to novelties in dairy products. The data were collected within a CAPI (Computer Assisted Personal Interview) survey on a representative sample of 983 adult Polish consumers. The non-hierarchical K-means clustering method was used to identify four clusters of consumers, namely: Enthusiastic, Involved, Ultra-involved and Neutral. Enthusiastic consumers attach more importance to the influence of dairy products on immunity and are more willing to agree with the opinion that dairy products are a source of mineral nutrients as well as vitamins. Ultra-involved and Involved consumers pay less attention to some health aspects of dairy products compared to other clusters; however, the Ultra-involved are more quality-oriented than are the Involved. Neutral consumers are more open to accept changes on the dairy product market and are relatively more inclined to choose new dairy products. However, these consumers have scored lower on those aspects related to the healthiness of dairy products and, in order to target them effectively, it is essential to develop well-tailored communication strategies highlighting the health benefits of dairy products. These results relate to the Polish market and are important for the development of new dairy products and for targeting public nutrition as well as for directing marketing communication. The results may provide important insights for those who develop educational strategies and campaigns.

## 1. Introduction

Dairy products are characterized by an appropriate nutritional value which contributes positively to human health [1,2,3]. Dairy products also play a key role in the prevalence and/or treatment of some diseases; examples include obesity [4,5,6], hypertension [7,8], type 2 diabetes [5,9,10], and cardiovascular disease [5,11,12,13]. Some kinds of dairy products are valued for their favorable effect on the digestive system [14,15]. Moreover, the consumption of dairy products is associated with lower body weight and/or body fat [16,17,18]. In general, the consumption of dairy products provides various health benefits [2]; however, some study results also show that, besides consumption-related benefits for example flavoring milk with added sugar may promote milk intake, this consumption may not be without adverse effects in terms of caloric intake and possibly obesity for children and adolescents [19]. Results of other studies also show that the possibility that milk intake is simply a marker of diets with good nutritional quality cannot be excluded, and this aspect needs further research [20].

The food sector faces an increasingly competitive and more globalized market and more demanding consumers who exhibit greater concern about quality and health benefits with respect to products [21]. Among the different product sectors, the dairy sector is the one that has undergone the greatest change, with the introduction of new products claiming healthy characteristics [22]. Polish consumers are increasingly interested in healthiness and the safety of food [23,24], and health is perceived as the most important value [25,26]. Consumer opinions on health are crucial, particularly when it comes to benefits and risks to health and disease prevention [27,28]. In addition, the effect on health is important in consumers’ choice behavior concerning dairy products [29].

Therefore, the aims of the current study were: (a) to identify consumer segments based on the importance they attached to selected attributes of dairy products, (b) to explore differences between the identified segments in their perceptions of health-related attributes of dairy products, (c) to determine if health-related aspects influenced consumers decision to buy high quality dairy products, and (d) to identify if consumers were open to novelties in dairy products.

## 2. Materials and Methods 

### 2.1. Data Collection Process

Quantitative data were collected in 2013 within the project “BIOFOOD-Innovative, Functional Products of Animal Origin” aimed at increasing innovation in the Polish agro-food sector through the development of products of animal origin providing functional and nutritional benefits. This paper presents some of the findings from a larger multidisciplinary study identifying drivers of and obstacles to innovation in products of animal origin [30], including consumer perception and acceptance of innovative food products [31]. 

The sample in our study (*N* = 983) was drawn from the Social Security addresses database and was representative for the national population in terms of age, gender and the region that consumers lived in. The survey was conducted in each of the 16 voivodships in Poland. After drawing the starting addresses, the random route method was used in the selection of the sample [32,33]. A number of sampling points were drawn with probability proportional to population size, for total coverage of the country, and to population density. In order to do so, the sampling points were drawn systematically from each of the “administrative regional units”, after stratification by individual unit and type of area. They thus represent the whole of Poland as well as the distribution of the resident population. In each of the selected sampling points, a starting address was drawn at random. Further addresses were selected by standard “random route” procedures from the initial address. In each household, a respondent was drawn, at random (following the “closest birthday rule”).

The interviews were conducted face-to-face at respondents’ homes by a professional market research agency in accordance with the ESOMAR (European Society for Opinion and Marketing Research) code of conduct using the CAPI (Computer Assisted Personal Interview) technique. All respondents were aged 21+. Only those respondents who met the recruitment criteria, i.e., made their own or cooperative food purchases and declared dairy product consumption, participated in the study.

### 2.2. Description of Questionnaire

The questionnaire used in the study was structured in two main blocks and covered aspects such as consumer perception of food quality and consumer perception of health aspects of dairy products. In order to identify consumer segments, a question related to the importance of different attributes of dairy products in consumer food choices was used for the factor and then cluster analysis (“Please specify to what extent the factors listed below encourage you to consume dairy products”). A 7–point scale was used, where 1–meant “definitely does not encourage to consume dairy products”, and 7–“definitely encourages you to consume dairy products”. Analysis of statements’ reliability in a given question was performed using Cronbach Coefficient Alpha. The obtained value of Cronbach Coefficient Alpha = 0.803 confirmed the right choice of questions for factor analysis (Principal Component Analysis–PCA). To profile segments, three further questions were used regarding opinions on dairy products. One of these referred to opinions on selected attributes of products. The next question identified opinions on high-quality products, including health aspects. In the following question, a scale designed for the needs of other studies on products of animal origin was used [34], including statements from other scales used in available literature on the subject [35,36]. The question was worded as follows: “People have different opinions on food. Please specify to what extent you agree or disagree with the statements on dairy products listed below”. For these 3 questions, a 7–point scale was used, where 1–meant “I strongly agree”, and 7–“I strongly disagree”. Analyses were conducted using the SAS 9.4 statistical package (SAS Institute, Cary, NC, USA).

## 3. Results

Consumer segmentation was preceded by a factor analysis using PCA with a varimax rotation of 14 statements. Based on eigenvalues (eigenvalues higher than 1), a 3–factor solution describing the phenomenon analyzed was suggested (Table 1). The factors obtained via PCA analysis explained 54.54% of total variation. Qualification for particular factors was based on the minimum value of factor loadings estimated at 0.4. Factor adequacy for the requirements of factor analysis, as studied by the Kaiser-Mayer-Olkin measure (KMO). The KMO value indicating collective correlation of variables was 0.89, which clearly confirmed the logic behind using the variable reduction method. Identified factors presented in Table 1 were used for cluster analysis (segmentation).

The division of consumers into segments was conducted in two stages. Firstly, a cluster analysis using hierarchical methods was performed. In the second stage of consumer segmentation, a cluster analysis was used based on non-hierarchical method K-means with initial cluster seeds obtained through the hierarchical method. The non-hierarchical K-means clustering method led to the identification of four clusters: Enthusiastic, Ultra-Involved, Involved, and Neutral. In general, the names of the clusters related to consumers’ perceptions of dairy products and reflected their mean scores; the highest for the Enthusiastic, average for the Ultra-Involved, relatively lower for the Involved and the lowest for the Neutral. The profiles of the resulting segments were determined using chi square cross-tabulation and ANOVA (Analysis of Variance) with post-hoc Waller-Duncan K-ratio *t* Test comparison of mean scores. The socio-demographic characteristics, including gender, age, education, place of residence, number of members in the household, number of children in the household, the subjective evaluation of health and income satisfaction, are presented in Table 2. The results of cluster analysis, together with the size of each cluster, are reported in Table 2, Table 3, Table 4 and Table 5. The data analysis presented in Table 2 indicates that, in the sample tested, there were statistically significant differences observed in terms of all the clusters identified. In the total sample, a slight majority of respondents were women. In terms of the age of the total sample, it can be seen that the largest age group was 45–54, people aged 65–75 were the least numerous. Over 40% of the surveyed declared the lowest education level, and people with university education were the least numerous group. Over 1/3 of the respondents declared living in rural areas, while every 10th respondent indicated living in big cities with over 500 thousand inhabitants. Almost 75% respondents lived in households comprising 2–4 persons, and over half of the people surveyed declared having children. A subjective evaluation of income satisfaction showed that almost 40% of the surveyed people lived frugally and had enough money to buy what they needed and over 1/3 lived very frugally to save money for major purchases. About half of the surveyed people evaluated their health as good, more than 20% as very good, and almost one quarter of respondents selected a neutral answer (i.e., “neither good nor bad”).

Cluster 1 (Enthusiastic–29.09%) was dominated by women and people aged 45–54, 55–64 as well as 35–44. Over 2/5 respondents were those with the lowest level of education, over 1/3 declared secondary education, and one fifth of respondents had a university education. The most numerous group were inhabitants of rural areas. A subjective evaluation of health showed that half of the respondents described their health as good and almost 20% as very good. A subjective evaluation of income satisfaction showed that the majority of the surveyed people lived frugally and had enough money or lived very frugally to save money for major purchases. Two- and three-person households were the most numerous in segment 2. Families with children constituted over half of this segment.

Cluster 2 (Ultra-involved–18.82%) was dominated by men, people aged 45–64 and by people with secondary or lower education levels, one fifth of respondents, similar to cluster 2, declared a university education; the biggest group of respondents came from rural areas. More than half of the people in this segment assessed their health as good and almost 30% as very good. A subjective evaluation of income satisfaction showed that over 2/5 of the surveyed people lived frugally and had enough money to buy what they needed. Compared with other segments, the largest share of people who have children was noted in segment 3.

Cluster 3 (Involved–27.56%) was dominated by people with lower education levels (primary, lower secondary and vocational) living in rural areas, small towns (up to 100 thousand inhabitants) and medium-sized cities (200–500 thousand inhabitants). In this segment, almost half of the people were those respondents who assessed their health as good, more than 25% assessed it as very good. Over 1/3 of this segment was constituted by people from two-person households. In this segment there was the lowest share of people with children, compared with other segments.

In cluster 4 (Neutral–24.41%) half of the people were respondents with the lowest levels of education (primary, lower secondary and vocational). In this segment, over 1/4 of those surveyed were people living in rural areas, and almost 60% of the people came from towns of over 20 thousand inhabitants (the aggregate of percentages for towns of 20 thousand–100 thousand, 100 thousand–500 thousand and of more than 500 thousand). More than 50% of the surveyed people from this segment assessed their health as good, and one tenth of respondents assessed it as very good. A subjective evaluation of income satisfaction showed that more than 2/5 of the surveyed people lived very frugally to save money for major purchases, and almost 1/3 lived frugally and had enough money to buy what they needed. The most represented households in this segment were 2-and 3-person ones. Moreover, an average share of people with children was noted among the Neutral group (Table 2). 

### 3.1. Consumer Perception of Health-Related Attributes of Dairy Products

Regarding opinions on perception of dairy products, respondents declared the highest level of agreement with the statement that dairy products are a rich source of protein and that dairy products, particularly yogurts, kefirs and butter milk, have a beneficial influence on immunity. In all the statements analyzed (Table 3), statistically significant (*p* < 0.05) differences were noted between mean scores in particular clusters. An additional post-hoc test (Waller-Duncan K-ratio *t* Test) compared mean values of opinions between pairs of clusters.

When compared to other segments, the Enthusiastic attached the most importance to the influence of dairy products on immunity (dairy products, particularly yogurts, kefirs and butter milk have a beneficial influence on immunity). They have agreed to a greater extent with the opinion that dairy products are a rich source of minerals as well as vitamins, and that products with reduced fat content are the best for one’s health. The Ultra-involved, to a greater extent than two other segments (i.e., Involved and Neutral), agreed with the statement that dairy products have a beneficial influence on immunity, that they are a source of minerals and vitamins and that healthy nutrients in dairy products have a beneficial influence on the digestive system.

The Involved, more often than the Neutral, agreed with the opinion that dairy products are a rich source of protein and have a beneficial influence on immunity and that dairy products are a rich source of minerals as well as vitamins. Moreover, the Neutral, more often than the Involved, agreed with the opinion that dairy products with reduced fat content are the best for one’s health. 

In all the analyzed statements on health aspects of dairy products, statistically significant (*p* < 0.05) differences were noted between mean scores in particular clusters. 

### 3.2. Quality Attributes of Dairy Products as Drivers of Food Purchasing Decisions

The degree of agreement with the statement on the importance of dairy product quality in the choice of food shows its significant role in consumers’ decisions, whereby the Enthusiastic agreed with it to the highest extent and Neutrals to the least extent (Table 4).

Compared with other clusters, the Involved to a lesser extent expressed their agreement with the statement that they bought high-quality dairy products due to their beneficial influence on children’s health or that they bought high-quality dairy products due to their beneficial influence on their body shape. The Neutral, more often than the respondents from other clusters, agreed with the opinion that they bought high-quality dairy products only for those family members who had health issues.

### 3.3. Consumers’ Willingness to Accept Innovation in Dairy Products

In order to identify respondents’ opinions on their willingness to buy dairy products and to accept changes on the food market regarding dairy products, those surveyed were asked about the level of their agreement with statements describing selected opinions on the willingness to buy dairy products and to accept some changes on the dairy product market. In all the analyzed statements on openness to new milk-based products, statistically significant differences were noted between mean scores in particular clusters (Table 5). An additional post-hoc test (Waller-Duncan K-ratio *t* Test) compared mean values of opinions between pairs of clusters.

The highest degree of agreement amongst respondents was noted for the statement: “If I do not know what is in a food, I will not try it”, “I am very particular about the foods I will eat”. Analysis of the responses in individual clusters showed that, compared with other consumers, the Neutral expressed the highest level of agreement with the majority of suggested statements concerning openness to new products, which may indicate that they exhibit greater pro-innovative attitudes. The Enthusiastic, to a greater extent than the Ultra-involved, declared that they usually know more than others about the latest products, they seek information on new dairy products appearing on the market and that they have concerns about eating dairy products they have not tried before. Compared with consumers from other clusters, the Involved declared a lower level of agreement with the majority of statements, which indicates the lowest level of openness to changes (innovation) on the dairy product market. Regarding concerns about eating products which have not been tried before, the Involved declared a relatively high level of agreement, a similar level to the Neutral respondents (Table 5).

## 4. Discussion

Our findings show that, compared with other consumers, the Enthusiastic assessed dairy products the most favorably due to their influence on immunity and their mineral and vitamin content. This group demonstrated the highest levels of agreement with the statement that dairy products with reduced fat content are the best for one’s health. The segment of the Enthusiastic comprised a higher share of women and mature respondents that exhibit higher health concerns and better knowledge on health-related aspects of food.

Female consumers show high acceptance for some functional dairy products, such as yogurt enriched with calcium, fiber and probiotics. Acceptance of functional dairy products increases amongst consumers with higher diet/health-related knowledge, as well as with age [37]. When it comes to fat content, for the choice of calorie reduced dairy products the highest priorities were: a low fat content, healthiness and good taste [38]. Moreover, low-fat dairy products, when consumed regularly as part of a balanced diet, may have a number of beneficial outcomes for neurocognitive health during ageing [39]. Furthermore, the main reason for choosing functional fermented dairy products is prevention of diseases, followed by the positive impact of low-fat content on body weight [40]. 

Agreement with the opinion that dairy products are a rich source of protein observed in our study was equally significant for the Enthusiastic and Ultra-involved. In general, milk proteins are precursors of bioactive peptides, so dairy products (e.g., fermented products) may be regarded as functional foods and targeted for imparting several biologically beneficial attributes [41]. Moreover, from the consumers’ point of view, products enriched with proteins could be beneficial for the elderly [42] as well as for people on special dietary regimes and those with certain health issues [43]. Results of other studies show that, for example, the use of protein-enriched drinking yogurt, consumed as part of regular meals, can be a promising and feasible solution to increase the protein intake of ill patients [44].

The results of our study showed that quality is the most important factor in choosing dairy products for the Enthusiastic, and the least important for the Neutral. It must, however, be added that consumers perceive the notion of ‘quality’ in a complex way. From the consumers’ perspective, there are many aspects which can describe the quality of a food product, i.e., intrinsic qualities (e.g., taste), as well as external factors (e.g., origin or labeling) [45,46]. Moreover, from the consumers’ point of view, the factors of dairy product quality include health and process-related quality that are credence dimensions. The above-mentioned quality components must be clearly communicated to a consumer to establish trust in them [47]. In our own studies respondents exhibited concerns about dairy products which they had not previously consumed, which is supported by high levels of agreement with the statements: “If I do not know what is in a food, I will not try it” and “I am very particular about the foods I will eat”. These opinions may also confirm the willingness to seek information, for example, about the composition of products in this category, which has also been noted in other studies [29,48].

Our research revealed that consumers’ choice of high-quality dairy products for their influence on children’s health was the most important factor for the following segments: Enthusiastic, Ultra-involved and Neutral. In the case of the first two segments, it is connected with the biggest share of families with children in these segments, which is confirmed in other studies [49]. As shown in the literature of the subject, dairy products contain many of the nutrients which are responsible, amongst other things, for the proper growth of children [50]. When it comes to yogurts [51], their frequent consumption, for example amongst American children, is associated with better diet quality, lower levels of fasting insulin, reduced insulin resistance scores and improved insulin sensitivity scores. 

Our own findings show that agreement with the opinions about buying high-quality dairy products for those family members who have health issues is the highest amongst Neutral respondents. The literature on the subject concerning health aspect of dairy products emphasizes that various types of dairy products have different importance levels for health, which is shown by the so-called risk markers of cardiovascular disease [52]. In addition, for health reasons it is advisable to consume dairy products with low fat content rather than products with high fat content [53]. The intake of milk and yogurts should be increased and the intake of cheese should be decreased [54]. However, a growing interest of consumers in the development of new functional foods and their introduction into healthy diets has been observed [15]. Functional dairy products will still play a significant role in a healthy diet [55]. 

Compared with other segments, the Neutral placed greater importance on the influence of body shape aspect, which can indicate that this group, to a greater extent, may be motivated by hedonic values and, therefore, exhibit greater concern for their appearance, which has also been noted in other studies [56].

As noted earlier, our own studies showed that the Neutral, compared with other consumers, were the least concerned about selected attributes of dairy products, such as their protein content, influence on immunity or presence of vitamins and minerals. However, the attributes discussed were rated relatively highly in the opinion of people in this segment, which could indicate that they matter during the decision-making process. Moreover, the Neutral, compared with other segments, showed the greatest acceptance for changes on the dairy food market and were relatively more willing to declare choosing new products. Consequently, this segment can be a potential group of people interested in new products on the market, including health-promoting food products. Moreover, other studies [57] have shown that health-promoting food products are important for consumers and the importance of the health aspect as the criterion for food choice is growing [34]. However, the Neutral segment does not possess characteristic socio-demographic attributes typical of innovative consumers. Other studies have shown that younger consumers declare higher acceptance for the suggested changes in products of animal origin [34,58,59]. The acceptance for changes also concerns, to a greater extent, better educated people [34,58,59] and those of higher income [34,60]. However, the Neutral segment is dominated by those living in cities, including the biggest cities, i.e., with over 500 thousand inhabitants. Findings from other authors [59,60,61] have shown that this group exhibits the greatest acceptance for changes to food including changes to products of animal origin [34,58]. Furthermore, big cities offer consumers more choice of products, which has been reported by other authors [62], who claim that small towns offer consumers a significantly narrower range of available goods.

## 5. Conclusions

Growing interest in health-related attributes of food has given rise to a new range of foods and products on the market that, as well as providing nourishment, improve health by increasing well-being and reducing the risk of certain diseases. Our research has revealed that consumers appreciate health-promoting attributes of dairy products declaring their use for a specific purpose and target group, i.e., for children or family members with health issues. Neutral respondents exhibited greater acceptance of changes in the dairy food market, which could indicate that this will be the group of people particularly interested in new products. However, they scored lower on those aspects related to the healthiness of dairy products and to target them effectively it is essential to develop well-tailored communication strategies highlighting the health benefits of dairy products.

Our results need to be repeated under different socio-cultural conditions and/or in other countries. 

These results are of relevance for professionals involved in public nutrition issues as well as for marketers aiming at development of well-tailored communication strategies. The results may provide important insights for those who develop educational strategies and campaigns. 

## Figures and Tables

**Table 1:** Principal components analysis (PCA) of consumers’ use of dairy product consumption factors; varimax rotated factor loadings percentage of explained variance (*N* = 983, Poland).

Food Characteristics	Factor 1 Hedonic Aspects	Factor 2 Health Aspects	Factor 3 Health Concerns
Easy to prepare	0.754		
A quick snack which can be eaten between meals	0.711		
Taste	0.696		
Wide product range	0.691		
Variety to the menu	0.661		
Habits	0.579		
Mineral and vitamin content	0.538		
Medical/dietary recommendations		0.797	
Appropriate animal breeding		0.675	
Health reasons		0.617	
Low level of processing		0.549	
Presence of preservatives			0.804
Presence of flavors			0.704
Fat content			0.447
The variance explained/% explained variance	34.79	10.66	9.09

**Table 2:** Socio-demographic characteristics of the consumers surveyed (*N* = 983, Poland).

Variables	Total Sample (%)	Cluster 1 Enthusiastic *N* = 286	Cluster 2 Ultra-Involved *N* = 185	Cluster 3 Involved *N* = 271	Cluster 4 Neutral *N* = 240	*p*-Value for 𝜒^2^ Test
Gender						0.0185
Women	50.97	58.04	44.32	50.92	47.72	
Men	49.03	41.96	55.68	49.08	52.28	
Age (years)						0.0182
21–27	16.3	11.19	22.16	17.34	16.6	
28–34	15.9	13.64	17.3	15.5	17.84	
35–44	18.3	17.83	20.54	16.61	19.09	
45–54	20.2	22.73	21.62	18.82	17.84	
55–64	18.4	19.58	12.43	22.14	17.43	
65–75	10.89	15.03	5.95	9.59	11.2	
Education						0.011
Vocational	46.80	44.41	41.08	50.18	53.53	
Secondary	37.81	36.36	39.46	36.53	37.34	
Higher	15.39	19.23	19.46	13.28	9.13	
Place of residence						<0.0001
Rural area	35.62	37.41	52.97	29.52	26.81	
Cities up to 20,000	13.51	13.99	9.73	14.76	14.47	
Cities above 20,000 to 100,000	19.96	18.53	16.22	20.3	24.26	
Cities above 100,000 to 500,000	19.75	19.23	14.05	24.72	19.15	
Cities above 500,000	11.16	10.84	7.03	10.7	15.32	
Number of persons in the household						0.0056
1	13.10	11.87	11.36	12.25	16.96	
2	27.71	28.06	21.59	31.62	27.68	
3	25.56	27.34	25.57	21.34	28.13	
4	21.70	21.22	21.02	22.92	21.43	
5 and more	11.92	11.51	20.45	11.86	5.8	
Children (Yes)	52.74	53.50	63.46	45.87	50.81	0.0087
Subjective assessment of financial situation						0.0155
Sufficient budget without necessity to economize	8.33	7.04	13.1	6.53	8.3	
We live frugally and have enough money to buy what we need	38.8	39.63	44.05	42.04	30.57	
We live very frugally to save money for major purchases	35.8	34.44	28.57	34.29	44.1	
We have enough money for the cheapest food or clothes and less	17.10	18.89	14.29	17.14	17.03	
Health assessment						0.0008
Very good	21.28	19.23	29.19	25.46	12.92	
Good	50.81	50.0	51.35	49.08	53.33	
Neither good nor bad	23.93	25.52	18.92	20.66	29.58	
Bad	3.56	4.9	0	4.06	4.17	
Very bad	0.41	0.35	0.54	0.74	0	

**Table 3:** Profile of the segments in terms of different perceptions of dairy products (*N* = 983, Poland).

Statements	Mean	Cluster 1 Enthusiastic *N* = 286	Cluster 2 Ultra-Involved *N* = 185	Cluster 3 Involved *N* = 271	Cluster 4 Neutral *N* = 240	*p*-Value
Dairy products are a rich source of protein	6.13	6.61 ^a^	6.52 ^a^	6.05 ^b^	5.35 ^c^	<0.0001
Dairy products, particularly yogurts, kefirs and butter milk, have a beneficial influence on immunity	5.85	6.33 ^a^	6.09 ^b^	5.68 ^c^	5.28 ^d^	<0.0001
Dairy products are a rich source of minerals	5.76	6.28 ^a^	6.04 ^b^	5.54 ^c^	5.18 ^d^	<0.0001
Dairy products are a rich source of vitamins	5.73	6.17 ^a^	5.95 ^b^	5.58 ^c^	5.19 ^d^	<0.0001
Healthy nutrients in dairy products have a beneficial influence on the digestive system	5.61	6.13 ^a^	5.94 ^a^	5.28 ^b^	5.10 ^b^	<0.0001
Dairy products with reduced fat content are the best for health	4.74	5.14 ^a^	4.53 ^b,c^	4.32 ^c^	4.84 ^a,b^	<0.0001

^a,b,c,d^ Means with the same letter are not significantly different; ANOVA (Analysis of Variance) post-hoc Waller-Duncan K-ratio *t* Test.

**Table 4:** Profiles of the segments in terms of different perceptions of high quality dairy products (*N* = 983, Poland).

Statements	Mean	Cluster 1 Enthusiastic *N* = 286	Cluster 2 Ultra-Involved *N* = 185	Cluster 3 Involved *N* = 271	Cluster 4 Neutral *N* = 240	*p*-Value
Quality matters to me while choosing dairy products	5.71	6.22 ^a^	5.98 ^b^	5.48 ^c^	5.14 ^d^	<0.0001
I buy high-quality dairy products because they have a beneficial influence on my children’s health	4.59	4.95 ^a^	4.63 ^a^	3.96 ^b^	4.78 ^a^	<0.0001
I buy high-quality dairy products because they have a beneficial influence on my body shape	4.08	4.39 ^a,b^	4.07 ^b^	3.32 ^c^	4.51 ^a^	<0.0001
I buy high-quality dairy products only for those family members who have health issues	3.18	3.12 ^b^	2.57 ^c^	2.82 ^b,c^	4.07 ^a^	<0.0001

^a,b,c,d^ Means with the same letter are not significantly different; ANOVA post-hoc Waller-Duncan K-ratio *t* Test.

**Table 5:** Profiles of the segments in terms of different perceptions referring to openness to buy new products and changes in the market of dairy products (*N* = 983, Poland).

Statements	Mean	Cluster 1 Enthusiastic *N* = 286	Cluster 2 Ultra-Involved *N* = 185	Cluster 3 Involved *N* = 271	Cluster 4 Neutral *N* = 240	*p*-Value
I like to buy new and different products	3.85	3.86 ^b^	3.90 ^b^	3.33 ^c^	4.38 ^a^	<0.0001
New products excite me	4.00	4.17 ^a,b^	3.92 ^b^	3.54 ^c^	4.37 ^a^	<0.0001
I try new products before my friends and neighbors	2.80	2.69 ^b^	2.46 ^b^	2.12 ^c^	3.93 ^a^	<0.0001
I know more than others about the latest new products	2.83	2.71 ^b^	2.34 ^c^	2.13 ^c^	4.03 ^a^	<0.0001
I look for information about what new food appears on the market	2.79	2.67 ^b^	2.35 ^c^	2.22 ^c^	3.93 ^a^	<0.0001
I am usually amongst the first to try new products	3.22	3.11 ^b^	3.03 ^b^	2.58 ^c^	4.17 ^a^	<0.0001
I am constantly sampling new and different foods	3.36	3.25 ^b^	3.11 ^b,c^	2.88 ^c^	4.20 ^a^	<0.0001
I do not trust new foods	3.79	3.69 ^b,c^	3.79 ^b^	3.40 ^c^	4.30 ^a^	<0.0001
If I do not know what is in a food, I will not try it	4.55	4.81 ^b^	4.34 ^b^	4.52 ^a,b^	4.43 ^a,b^	0.0461
I like foods from different countries	3.29	3.3 ^b^	3.22 ^b^	2.57 ^c^	4.09 ^a^	<0.0001
Ethnic food looks too weird to eat (e.g., Asian cuisine)	3.72	3.56 ^b^	3.5 ^b^	3.42 ^c^	4.31 ^a^	<0.0001
At dinner parties, I will try a new food	3.64	3.81 ^b^	3.51 ^b^	2.99 ^c^	4.25 ^a^	<0.0001
I am afraid to eat things I have never eaten before	3.90	3.69 ^b^	3.34 ^c^	4.15 ^a^	4.31 ^a^	<0.0001
I am very particular about the foods I will eat	4.48	4.68 ^a^	4.57 ^a,b^	4.23 ^b^	4.45 ^a,b^	0.0331
I will eat almost anything	4.10	3.99 ^b^	4.39 ^a^	3.72 ^b^	4.43 ^a^	0.0003
I like to try ethnic restaurants (e.g., Asian cuisine)	2.92	2.56 ^b^	2.75 ^b^	2.58 ^b^	3.85 ^a^	<0.0001

^a,b,c^ Means with the same letter are not significantly different; ANOVA post-hoc Waller-Duncan K-ratio *t* Test.
